# *Mycobacterium bovis* Infection in Holstein Friesian Cattle, Iran

**DOI:** 10.3201/eid1412.070727

**Published:** 2008-12

**Authors:** Keyvan Tadayon, Nader Mosavari, Fardin Sadeghi, Ken J. Forbes

**Affiliations:** University of Aberdeen, Foresterhill, Aberdeen, Scotland, UK (K. Tadayon, K.J. Forbes); Hesarak Institute, Karaj, Iran (K. Tadayon, N. Mosavari); Iranian Veterinary Organisation, Tehran, Iran (F. Sadeghi)

**Keywords:** Mycobacterium bovis, bovine tuberculosis, RD typing, spoligotyping, VNTR typing, Holstein Friesian, cattle, Iran, dispatch

## Abstract

To identify strains of *Mycobacterium bovis* circulating in Iran, we used region of difference, spoligotypes, and variable number tandem repeats to genotype 132 *M. bovis* isolates from Holstein Friesian cattle. Despite wide geographic origins, the strains were genetically homogeneous. Increased distribution of cattle herds and inadequate control measures may have contributed to strain dispersion.

Estimates suggest that globally >50 million cattle are infected with *Mycobacterium bovis*, causing an annual loss of ≈$3 billion US (*1*). In Asia, 94% of the 460-million cattle herd (33% of the world’s cattle) are in areas with either no or only partial tuberculosis (TB) control programs (*2*). In 2006, the prevalence of bovine TB in Iran was 0.12% (Iranian Veterinary Organisation [IVO], unpub. data), yet few studies have been conducted on *M. bovis* in Iran (*3*–*5*). To identify the strains of *M. bovis* in Iran, we used region of difference (RD) typing, spoligotyping, and variable number tandem repeats (VNTR) typing.

## The Study

From 1996 through 2003, we collected necropsy specimens from TB-test reactor cattle from abattoirs in 21 of the 28 Iranian provinces where bovine TB has been reported. Specimens were all respiratory and gastrointestinal lymph nodes and any lungs, spleens, or livers that were visibly affected. All specimens were cultured for *M. tuberculosis* complex bacteria and incubated for >10 weeks. Of the 470 animals tested, results were positive for 216; however, because of delays in exporting samples to the United Kingdom, only 132 samples contained reculturable isolates with sufficient growth for DNA extraction. Molecular speciation was determined by RD-PCR (RD1, RD5, RD9, RD10, and RD11) (*6*). Spoligotyping was conducted according to the method of Kamerbeek et al. (*7*). VNTR-PCR was conducted according to the 6-locus method of Frothingham and Meeker-O’Connell (exact tandem repeat [ETR]-A through ETR-F loci) (*8*) plus QUB11B and VNTR3232 loci (*9*).

**Figure 1:**
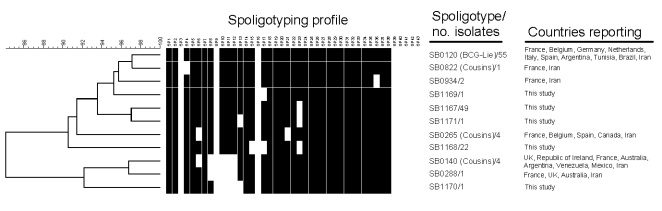
Spoligoprofiles of Iranian isolates and dendrogram of profile relatedness by the Dice and unweighted pair group method with arithmetic mean and previously published locations of profiles. The three spoligotypes from Iran were previously reported by Cousins et al. (*10*).

**Figure 2:**
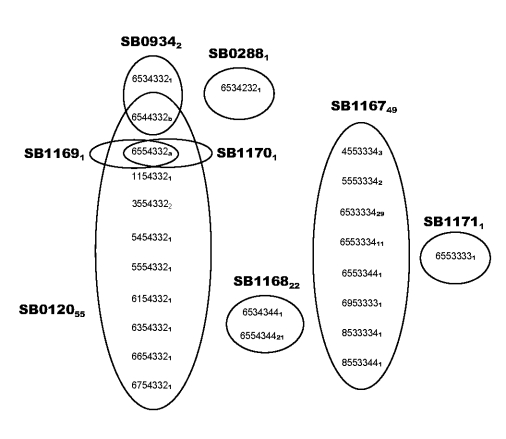
Variable number tandem repeat (VNTR) profiles and their abundance and distribution with spoligotypes. VNTR profiles are listed in the following order: exact tandem repeat (ETR)-A, B, C, D, E, F, QUB11B. The number of isolates of each spoligotype and of each VNTR profile are indicated by the subscript number; “a” indicates 42 SB0120 isolates, 1 SB1169 isolate, and 1 SB1170 isolate; “b” indicates 1 SB0120 isolate and 1 SB0934 isolate.

RD-PCR showed that all 132 isolates were *M.* *bovis*. Spoligotyping identified 8 types (Figure 1). SB0120 was the most common, and 5 others (SB1167–SB1171) were novel patterns and, thus, were specific to Iran. VNTR typing identified 23 profiles (Figure 2).

## Conclusions

RD typing of the 132 isolates confirmed that they were all wild type *M. bovis;* none were the *M. bovis* BCG vaccine strain because they carried the RD1 region. This finding is noteworthy because unauthorized vaccination of cattle with BCG has been reported in Iran (IVO, unpub. data). Although previous studies in Iran have reported the isolation of *M. tuberculosis* from tuberculin-positive cattle (*3*), our RD9 and RD10 analyses indicated that no isolates were *M. tuberculosis.* This finding suggests that *M. tuberculosis* is unlikely to be abundant, if even present, on cattle farms of Iran. Similarly, RD5 and RD11 analyses indicated that no isolates were *M. africanum* or *M. microti.*

The spoligotypes were either identical to the BCG-like (SB0120) pattern (41% of isolates) or were simple variants of it by the deletion of 1 or occasionally 2 single or contiguous blocks of spacers (Figure 1). Because spoligotype changes have been attributed solely to the deletion of spacer units, the BCG-like strains here are believed to be ancestral (*11*).

VNTR typing of the 132 *M. bovis* isolates at 8 loci identified 23 different profiles (Figure 2), 4 of which represented 80% of the isolates. The homogeneity of *M. bovis* isolates in Iran was further exemplified by the low diversity seen at ETR-E (2 alleles, 1 allele represented by only 1 isolate) and VNTR3232 (1 allele); these findings contrast with findings of greater heterogeneity, particularly at VNTR3232, reported elsewhere (*12*). Given the large geographic area covered by cattle in the present study, this level of homogeneity was unexpected. This finding is paralleled in the United Kingdom, where ETR-E is virtually monotypic and is believed to indicate a minimal effect of penetrating exotic strains (*11*).

In combination, spoligotyping and VNTR typing stratified the 132 isolates into 26 groups (Figure 2). Most isolates with a particular VNTR profile were found to be a subset of isolates with a specific spoligotype. Thus, VNTR could be used to subtype isolates identified by spoligotyping; presumably because of the more rapid rate of polymorphism changes in VNTR than in spoligotype.

Spoligotyping and VNTR typing showed high similarities for all isolates. Such homogeneity, in combination with the geographic restriction of several of the spoligotypes to Iran (at least in current databases), does not easily support the hypothesis that most strains currently circulating in Iran have been imported from abroad. Since the introduction of European breeds in the 1930s, Iran’s cattle herd has expanded constantly; expansion during the past 4 decades has been ≈1.8% annually (IVO, unpub. data). Given the susceptibility of these European breeds to bovine TB and the initial absence of effective disease control, as the Holstein Friesian herd increased in number, infections with *M. bovis* likely increased in parallel. The homogeneity and localization of the *M. bovis* strains to Iran would be a direct consequence of this dramatic increase in number of bovine TB–susceptible cattle from what has effectively been a genetic bottleneck for *M. bovis*.

The subsequent test-and-slaughter program in Iran may have contributed to the clonality of the *M. bovis* population. This situation would be similar to that in the United Kingdom, where typing of *M. bovis* strains from human patients (presumably infected with *M. bovis* from cattle) suggests that *M. bovis* was more diverse 50 years ago than it is today (*13*,*14*). It is believed that bovine TB control measures throughout the United Kingdom over the past 100 years reduced the *M. bovis* population size and diversity and led to geographic localization of *M. bovis* strains (*9*,*11*). The lower heterogeneity of isolates in Iran perhaps reflects a shorter timescale of events there than in the United Kingdom. Spoligotypes are reported to change over timescales as long as 60 years (*15*); the expansion of the *M. bovis* population in Iran over ≈50 years and the generation of 2–3 sequential spoligotype changes during this time is certainly compatible with these timescales. The absence of geographic regionalization of strains in Iran may also reflect the shorter timescale of events in Iran than in the United Kingdom and insufficient time for significant diversification of new strains. The extensive movement of cattle around Iran would also be expected to reduce regionality of strains.

What then is the origin of the currently circulating strains in Iran? Some of the spoligotypes found in Iran have been reportedly found elsewhere in the world; however, given the simplicity of many of the profiles from the Iran strains, homoplasy may well account for these, usually rarer, spoligotypes. Of the 55 isolates with the SB0120 profile, 42 had a common VNTR profile (Figure 2), which suggests that this strain, or perhaps 1 of the VNTR variants, would have been the progenitor strain from Iran. Whether such an ancestral strain originated in Iran or had been imported into Iran is a yet-unanswered question.

In a relatively short time, *M. bovis* has emerged as a major cause of cattle illness and economic loss in Iran, notably as a result of the ever-increasing numbers of susceptible hosts. Other causes may be changes in farming practices, such as intensification, and the continued escape of *M. bovis* from the test-and-slaughter scheme, possibly as a result of selection for less easily detectable strains. Without strengthened control measures, *M. bovis* is unlikely to disappear. Indeed, more infective animals in a growing population of susceptible animals increase the risk for other species and for humans.
